# On Gout and Phthisis

**Published:** 1849-03

**Authors:** 


### PATHOLOGY AND PRACTICE OF MEDICINE.

*On Gout and Phthisis.*—At the Westminster Medical Society, November 18, 1848, Dr. Garrod read a paper on the simultaneous progress of cases of gout and phthisis. He was induced to bring this subject before the notice of the Society, as in a paper on phthisis, communicated during the last session, it was asserted that a gouty condition of the system or blood was inimical to the development of tubercular disease, and it was suggested that, for the purpose of preventing or curing the latter affection, an attempt should be made to produce a gouty diathesis; and even the internal administration of urate of soda was hinted at. Dr. Garrod first spoke of some recent researches he had made on the subject of gout, which will appear in the volume of the Transactions of the Royal Medico-Chirurgical Society now in the course of publication, and described what he considered to constitute a gouty condition of blood, alluding to the presence of an excess of uric acid, before and during the paroxysm, in acute gout, and as an almost constant accompaniment in those forms of the disease where tophaceous or chalk-like deposits take place in different parts of the body. Dr. Garrod then stated, that if the gouty and tubercular diathesis were antagonistic, phthisis would never become developed in the inveterate forms of gout above alluded to. To prove, however, the fallacy of the idea, the following case was related:—A young man, aged twenty-eight, a native of London, whose father and grandmother had suffered from gout, applied for relief at University College Hospital, and was admitted, under the care of Dr. Williams. He was a painter by trade, and for some years he had been of very intemperate habits, but until the last few, had had plenty of food and clothing. From the age of seventeen, he had suffered from what he termed "rheumatism," (gout?) but had had an affection of the heart with it. Formerly, he was of full habit, but about three years since, began to lose both flesh and colour, although he did not feel particularly ill, and had no cough at the time. He was soon after seized with an attack of gout, both in his feet and hands, tophaceous deposits formed, and he was confined to his bed for twenty-eight weeks. About two months after his recovery, he was again attacked, and then had a severe cough, with expectorations of a greenish hue. The pectoral symptoms continued for about four months, the gouty two months longer. From this date until his admission into the hospital he was constantly suffering from chest affection and gout; hæmoptysis had occurred once, and deposits of urate of soda frequently came way from his joints. When admitted into the hospital, he was pallid and emaciated; complained of pain in various joints arising from gouty inflammation; also of pain in his side, cough, and expectorations of a muco-purulent character. On physical examination, clear evidence was found of the existence of tubercular deposition in both lungs, especially the left, at the apex of which, a distinct cavity was indicated by the production of pectoriloquy and cavernous respiration; during the remaining month of his life, the gouty affection continued to progress, now appearing in one part, now in another, and occasionally with the discharge of urate of soda from some of the joints. The thoracic affection also continued to advance, accompanied with hectic symptoms, increase of cough, and sharp pain in different parts of the chest, until he fell into a state of stupor, and so continued for a day or so, when death took place. The post mortem appearances fully proved the accuracy of the diagnosis, At the apex of the right lung a cavity was found, large enough to contain a walnut, the rest of the lung being studded with scattered tubercles in different stages of development. The apex of the left lung was excavated to the depth of four or five inches, and the remaining portion was sprinkled throughout with grey tubercles. The heart was healthy; the liver had patches of soft tuberculous deposit on its surface; the kidneys were small, and many of the tubuli filled with a white matter, consisting of crystallized urate of soda and uric acid; spleen enlarged. Mucous membrane of the colon ulcerated in patches. An examination of the blood was also made, and it was found to contain a very large amount of uric acid, larger than Dr. Garrod had ever before obtained. Some remarks were then made on other cases, in which gout and phthisis existed together; the rarity of the combination being easily accounted for by the fact, that gout in general does not appear till after the age of forty, whereas, tubercular disease is much more frequent before that period. It also appeared very doubtful to the author whether, granting the correctness of the hypothesis advanced in the paper alluded to, a gouty condition of blood could be induced by the internal administration of urate of soda.—*Lancet.*

